# Comparison of Various Anthropometric Indices as Risk Factors for Hearing Impairment in Asian Women

**DOI:** 10.1371/journal.pone.0143119

**Published:** 2015-11-17

**Authors:** Seok Hui Kang, Da Jung Jung, Kyu Yup Lee, Eun Woo Choi, Jun Young Do

**Affiliations:** 1 Division of Nephrology, Department of Internal Medicine, Yeungnam University Hospital, Daegu, Republic of Korea; 2 Department of Otorhinolaryngology-Head and Neck Surgery, School of Medicine, Kyungpook National University Hospital, Daegu, Republic of Korea; Weill Cornell Medical College in Qatar, QATAR

## Abstract

**Background:**

The objective of the present study was to examine the associations between various anthropometric measures and metabolic syndrome and hearing impairment in Asian women.

**Methods:**

We identified 11,755 women who underwent voluntary routine health checkups at Yeungnam University Hospital between June 2008 and April 2014. Among these patients, 2,485 participants were <40 years old, and 1,072 participants lacked information regarding their laboratory findings or hearing and were therefore excluded. In total 8,198 participants were recruited into our study.

**Results:**

The AUROC value for metabolic syndrome was 0.790 for the waist to hip ratio (WHR). The cutoff value was 0.939. The sensitivity and specificity for predicting metabolic syndrome were 72.7% and 71.7%, respectively. The AUROC value for hearing loss was 0.758 for WHR. The cutoff value was 0.932. The sensitivity and specificity for predicting **hearing loss** were 65.8% and 73.4%, respectively. The WHR had the highest AUC and was the best predictor of metabolic syndrome and hearing loss. Univariate and multivariate linear regression analyses showed that WHR levels were positively associated with four hearing thresholds including averaged hearing threshold and low, middle, and high frequency thresholds. In addition, multivariate logistic analysis revealed that those with a high WHR had a 1.347–fold increased risk of hearing loss compared with the participants with a low WHR.

**Conclusion:**

Our results demonstrated that WHR may be a surrogate marker for predicting the risk of hearing loss resulting from metabolic syndrome.

## Introduction

Metabolic syndrome is a well-known risk factor for cardiovascular diseases including atherosclerosis and hypertension (HTN) and is diagnosed according to five criteria: increased waist circumference (WC), high triglyceride level, low high-density lipoprotein (HDL) cholesterol level, high blood pressure, and high fasting glucose level [1]. Obesity is a component of and risk factor for metabolic syndrome. Many indices such as body mass index (BMI), WC, waist to hip ratio (WHR), or waist to height ratio (WHtR) have been introduced as indicators for obesity, but there are controversies regarding the best predictive methods among these anthropometric indices.

Hearing loss is a major public health problem that is associated with a low quality of life [2,3]. The prevalence of sensorineural hearing loss will increase as the number of elderly individuals increases. Among various anthropometric indices related to obesity, some investigations showed associations between hearing impairment and BMI, WC, or WHR [4–9]. However, among various anthropometric indices, the appropriate choice for predicting hearing impairment remains unresolved.

The WHR is a well-known indicator of metabolic disturbance that is related to the amount of visceral fat, and a high WHR is associated with the overproduction of adverse adipokines and insulin resistance [10]. Therefore, a patient with a high WHR is more likely to develop metabolic disturbances such as diabetes mellitus (DM) or HTN. These metabolic disturbances result in vasculopathies in various small vessels including the stria vascularis, damage to which is associated with hearing impairments [11]. Our previous study showed an association between metabolic syndrome and hearing impairment [12]. Therefore, the WHR may be associated with hearing loss, and could provide a simple predictive measure for its early diagnosis in Asian women. In addition, in individuals with a high WHR, the diagnosis and correction of metabolic disturbances may help prevent the progression or development of hearing loss. The objective of the present study was to examine the associations between various anthropometric measures and metabolic syndrome or hearing impairment in Asian women.

## Methods

### Study population

**Fig 1:**
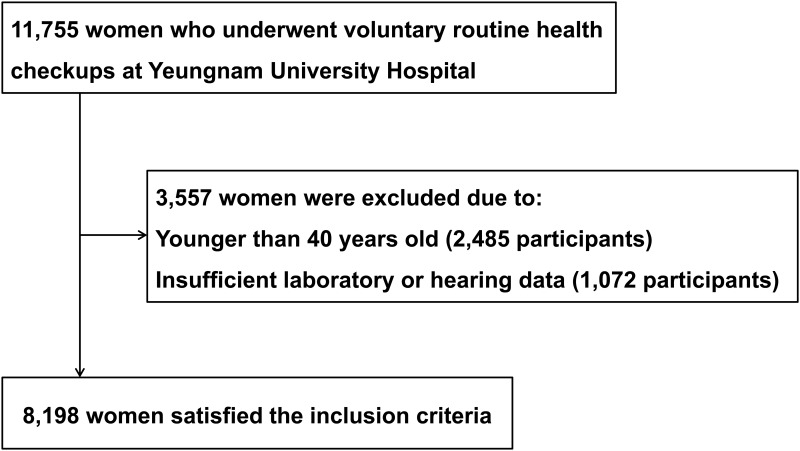
Study flow chart.

This was a single center-retrospective study. We identified 11,755 women who underwent voluntary routine health checkups at Yeungnam University Hospital between June 2008 and April 2014 (Fig 1). Participants from both rural and urban area visited our hospital, and routine health checkups with common screening examinations were performed regardless of the underlying disease or the area in which the patient lived. When a subject underwent multiple examinations, we analyzed the data acquired during their first visit. Among these patients, 2,485 participants were <40 years old, and 1,072 participants lacked information regarding their laboratory findings or hearing and were therefore excluded. In total 8,198 participants were recruited into our study. This study was approved by the Institutional Review Board of Yeungnam University Hospital (2015-06-003). The board waived the need for informed consent, as the subjects’ records and information were anonymized and de-identified prior to the analysis.

### Study variables

The subjects arrived at the hospital after an overnight fast. Clinical and laboratory data collected during the health examination included age, sex, hearing thresholds, BMI (kg/m^2^), WHR, WHtR, WC (cm), body shape index (BSI), body adiposity index (BAI, %), visceral fat area (VFA, cm^2^), systolic blood pressure (mmHg), diastolic blood pressure (mmHg), and fasting glucose (mg/dL), triglyceride (mg/dL), and HDL cholesterol (mg/dL) levels.

Fasting glucose, triglyceride, and HDL cholesterol levels were measured using the Hitachi Automatic Analyzer 7600 (Hitachi, Japan) by the enzymatic method (Sekisui Co., Japan). DM was defined as a self-reported history of a DM diagnosis or a fasting glucose level of ≥126 mg/dL. HTN was defined as systolic blood pressure of ≥140 mmHg, diastolic blood pressure ≥90 mmHg, self-reported history of HTN, or use of anti-hypertension drugs. Metabolic syndrome was defined according to the National Cholesterol Education Program Adult Treatment Panel III guidelines [1].

Weight, height, WC, and hip circumference were measured by well-trained medical professionals. Standing height was measured with the subject facing directly ahead with shoes off, feet together, arms by the sides, and heels, buttocks, and upper back in contact with the wall using a SECA 225 (SECA, Hamburg, Germany). Weight was measured using a GL-6000-20 scale (Cass, Seoul, Korea). WC was measured at the midpoint between the bottom of the rib cage and the top of the iliac crest. Hip circumference was measured over the widest part of the gluteal region. BMI was calculated by dividing the total body weight in kilograms by the square of the participant’s height in meters. WHR and WHtR were calculated as WC divided by hip circumference or height. BAI and BSI were calculated using equations described previously [13,14]:

$$\text{BAI}=\left[\frac{\text{hip circumference }(\text{m})}{\text{height }{\left(\text{m}\right)}^{1.5}}\right]-18$$

$$\text{BSI}=\frac{\text{WC }(\text{m})}{\text{BMI}{\;}^{2/3}\times \text{height }{\left(\text{m}\right)}^{1/2}}$$

VFA was measured using multi-frequency bioimpedance analysis (In-Body 720; Biospace, Seoul, Korea).

### Hearing assessment

Audiologic evaluation was performed by a specialist practitioner with training/supervision provided by a certified audiologist, which included on-site visits to ensure quality control. Air-conduction pure-tone thresholds were obtained in a soundproof booth using an audiometer (Audiometer, GSI 10 Audiometer GSI^®^, USA) and headphone (TDH-50p, Telephonics GSI^®^, USA). Testing was conducted as per the recommended standard audiometry procedures using automatic mode, except when respondents could not physically press the response button, or when there were difficulties using automatic mode. Manual mode was carried out using the down-10 up-5 method [15]. To avoid interference with audiometric evaluation, subjects were asked to refrain from chewing gum or eating candy.

The hearing thresholds were measured at 0.5, 1, 2, 3, 4, and 6 kHz. Many criteria for hearing thresholds have been used for hearing thresholds, including the use of different thresholds in each ear and a threshold based on the mean value of both ears [5,16,17]. In the present study, hearing thresholds were defined using the mean value of both ears. For each subject, the pure tone hearing thresholds were averaged at 0.5 and 1 kHz to obtain the Low-Freq value, at 2 and 3 kHz to obtain the Mid-Freq value, and at 4 and 6 kHz to obtain the High-Freq value. The pure tone average (PTA) was calculated as pure tone average at 4 frequencies (0.5, 1, 2, and 4 kHz). Hearing loss was defined by a PTA > 25 decibels (dB HL), in accordance with the American Speech-Language Hearing Association guidelines [18].

### Statistical analyses

Data were analyzed using SPSS version 21 (SPSS, Chicago, IL, USA) or MedCalc version 11.6.1.0 (MedCalc, Mariakerke, Belgium). Categorical variables are expressed as numbers and percentages, and continuous variables are expressed as the mean ± the standard deviation or standard error. We calculated the sensitivity, specificity, cutoff values, and probability of area under the receiver operating characteristic curve (AUROC) to predict metabolic syndrome or hearing loss using variable anthropometries indices. The best cutoff risk point was derived from the maximum of the Youden index in the AUROC. Correlation analysis was performed to assess the strength of the relationship between variable anthropometric indices and VFA or numbers of metabolic syndrome components.

Linear regression analysis was used to determine whether the WHR contributes to the hearing threshold, which is a continuous variable expressed as dB HL. The patients were divided into low and high WHR groups according to the cutoff value for hearing loss. Changes in hearing thresholds according to the two WHR groups were analyzed using an independent t-test for univariate analysis and analysis of covariance for multivariate analysis. Logistic regression analyses were used to determine whether the WHR contributes to hearing loss. Pearson’s χ^2^ test or Fisher’s exact test were used to assess the strength of association between age and the presence of metabolic syndrome or hearing loss. Multivariate analysis was performed using age, DM, HTN, and WHR. The level of statistical significance was set at *P* < 0.05.

## Results

### Baseline characteristics of participants

The mean age of the participants was 54.7 ± 9.9 years (range 40–88 years, Table 1 and S1 Fig), and 5,382 participants (65.7%) lived in an urban area (S2 Fig). The majority of patients (64.9%) lived in the metropolitan center of Daegu. There were 614 (7.5%) and 1,273 (15.5%) participants with DM or HTN, respectively. The AUROCs for metabolic syndrome of hearing loss were analyzed in participants. The mean hearing thresholds were 23.4 ± 11.0 dB HL for the Low-Freq threshold, 25.6 ± 13.5 dB HL for the Mid-Freq threshold, 30.1 ± 17.1 dB HL for the High-Freq threshold, and 24.4 ± 11.7 dB HL for the PTA.

**Table 1:** Clinical characteristics of participants.

Variable	Value
Age	54.7 ± 9.9
BMI (kg/m^2^)	23.4 ± 3.0
WHR	0.922 ± 0.055
WHtR	0.49 ± 0.05
WC (cm)	76.9 ± 7.8
BSI	0.075 ± 0.004
BAI (%)	27.9 ± 3.2
Triglyceride (mg/dL)	107.3 ± 69.6
HDL cholesterol (mg/dL)	60.7 ± 15.2
Fasting blood glucose (mg/dL)	92.8 ± 19.8
Systolic blood pressure (mmHg)	116.5 ± 15.4
Diastolic blood pressure (mmHg)	73.5 ± 11.0
Diabetes mellitus (%)	614 (7.5%)
Hypertension (%)	1,273 (15.5%)
VFA (cm^2^)	93.3 ± 25.3
Low-Freq (dB HL)	23.4 ± 11.0
Mid-Freq (dB HL)	25.6 ± 13.5
High-Freq (dB HL)	30.1 ± 17.1
PTA (dB HL)	24.4 ± 11.7

Abbreviations: BMI, body mass index; WHR, waist to hip ratio; WHtR, waist to height ratio; WC, waist circumference; BSI, body shape index; BAI, body adiposity index; HDL, high-density lipoprotein; VFA, visceral fat area; Low-Freq, low frequency; Mid-Freq, mid-frequency; High-Freq, high-frequency; PTA, pure tone average; dB HL, decibel.

Data are expressed as numbers (percentages) for categorical variables and mean ± standard deviation for continuous variables.

### Comparison of various indices for predicting metabolic syndrome or hearing loss

**Fig 2:**
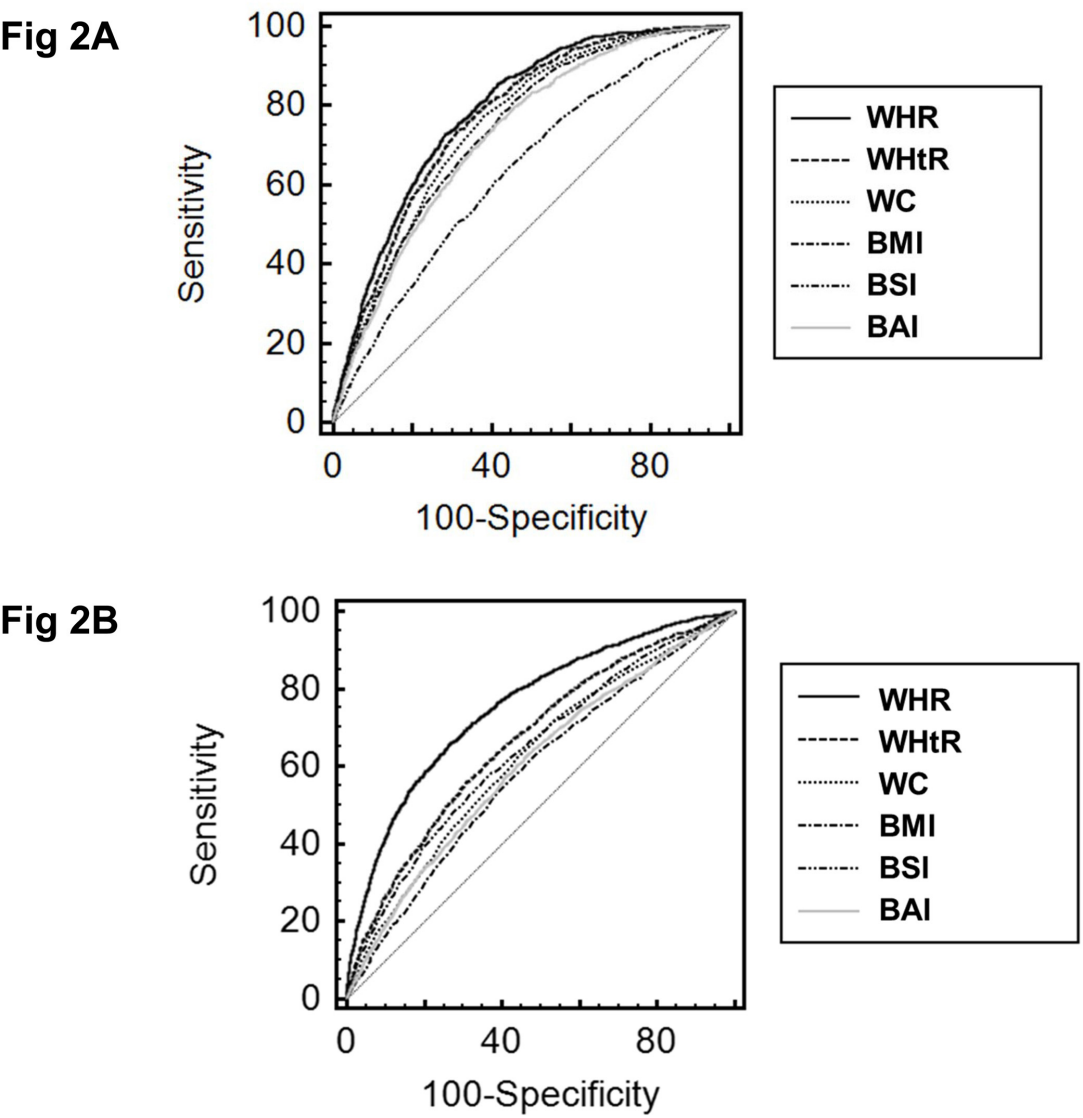
Receiver operating characteristic curves of anthropometric indices for the prediction of metabolic syndrome or hearing loss. A. Metabolic syndrome. B. Hearing loss. Abbreviations: WHR, waist to hip ratio; WHtR, waist to height ratio; WC, waist circumference; BMI, body mass index; BSI, body shape index; BAI, body adiposity index.

The AUROC value for metabolic syndrome was 0.790 (95% CI, 0.781–0.798) for WHR (Table 2 and Fig 2). The cutoff value was 0.939. The sensitivity and specificity for predicting metabolic syndrome were 72.7% and 71.7%, respectively. The AUROC value for hearing loss was 0.758 (95% CI, 0.748–0.767) for WHR. The cutoff value was 0.932. The sensitivity and specificity for predicting hearing loss were 65.8% and 73.4%, respectively. The WHR had the highest AUC and was the best predictor of metabolic syndrome and hearing loss.

**Table 2:** Comparison of AUROC for prediction of metabolic syndrome or hearing loss among variable indices.

Variables	Metabolic syndrome				Hearing loss			
	AUC (95% CI)	Cutoff	Sensitivity	Specificity	AUC (95% CI)	Cutoff	Sensitivity	Specificity
WHR	0.790 (0.781–0.798)	0.939	72.7%	71.7%	0.758 (0.748–0.767)	0.932	65.8%	73.4%
WHtR	0.773 (0.763–0.782)^a^	0.50	73.7%	68.5%	0.668 (0.658–0.678)^a^	0.50	55.9%	69.1%
WC	0.753 (0.744–0.763)^a^	77.5	76.9%	62.5%	0.622 (0.611–0.632)^a^	74.0	72.4%	46.2%
BMI	0.739 (0.730–0.749)^a^	23.0	81.3%	54.3%	0.589 (0.578–0.600)^a^	22.9	62.4%	52.3%
BSI	0.637 (0.626–0.647)^a^	0.075	64.4%	55.9%	0.641 (0.631–0.652)^a^	0.076	52.7%	68.3%
BAI	0.727 (0.717–0.737)^a^	27.9	76.3%	57.9%	0.606 (0.595–0.616)^a^	27.7	60.6%	55.8%

Abbreviations: AUROC, area under the receiver operating characteristic curve; AUC, area under the curve; CI, confidence interval; WHR, waist to hip ratio; WHtR, waist to height ratio; WC, waist circumference; BMI, body mass index; BSI, body shape index; BAI, body adiposity index.

^a^ *P* < 0.0001 compared with the WHR.

The AUCs of WHR were 0.698 (95% CI, 0.688–0.708) for elevated fasting glucose level, 0.727 (95% CI, 0.717–0.737) for elevated blood pressure, 0.688 (0.677–0.698) for elevated triglyceride level, and 0.643 (0.632–0.653) for decreased HDL cholesterol (Table 3). For elevated fasting glucose and elevated blood pressure, the WHR had the highest AUCs. For elevated triglyceride, the WHR and WHtR had higher AUCs compared with other indices. There was no significant difference between those two indices. For decreased HDL cholesterol, WHR, WHtR, WC, and BMI had higher AUCs compared with other indices. There were no significant differences between those four indices. Correlation analysis between anthropometric indices and VFA or numbers of metabolic syndrome components showed that WHR had the highest correlation with VFA and numbers of metabolic syndrome components (Table 4).

**Table 3:** The AUROC of anthropometric indices for the presence of metabolic syndrome components.

Variables	Elevated FG	Elevated BP	Elevated TG	Decreased HDL-C
	AUC (95% CI)	AUC (95% CI)	AUC (95% CI)	AUC (95% CI)
WHR	0.698 (0.688–0.708)	0.727 (0.717–0.737)	0.688 (0.677–0.698)	0.643 (0.632–0.653)
WHtR	0.682 (0.672–0.692)^b^	0.667 (0.657–0.677)^a^	0.691 (0.681–0.701)^c^	0.658 (0.648–0.668)^c^
WC	0.669 (0.659–0.679)^a^	0.640 (0.630–0.651)^a^	0.673 (0.663–0.683)^b^	0.644 (0.633–0.654)^c^
BMI	0.646 (0.635–0.656)^a^	0.644 (0.634–0.655)^a^	0.656 (0.646–0.666)^a^	0.635 (0.624–0.645)^c^
BSI	0.616 (0.605–0.627)^a^	0.573 (0.562–0.584)^a^	0.602 (0.591–0.612)^a^	0.578 (0.567–0.589)^a^
BAI	0.634 (0.624–0.645)^a^	0.648 (0.638–0.658)^a^	0.642 (0.631–0.652)^a^	0.619 (0.608–0.630)^b^

Abbreviations: AUROC, area under the receiver operating characteristic curve; FG, fasting glucose; BP, blood pressure; TG, triglyceride; HDL-C, high-density lipoprotein cholesterol; AUC, area under the curve; CI, confidence interval; WHR, waist to hip ratio; WHtR, waist to height ratio; WC, waist circumference; BMI, body mass index; BSI, body shape index; BAI, body adiposity index.

^a^ *P* < 0.0001 compared with the WHR.

^b^ *P* <0.05 compared with the WHR.

^c^ *P* >0.05 compared with the WHR.

**Table 4:** Correlation between anthropometric indices and VFA or number of metabolic syndrome components.

Variable	VFA	Number of metabolic syndrome components
	*r*	*r*
WHR	0.901	0.595
WHtR	0.823	0.571
WC	0.773	0.547
BMI	0.796	0.532
BSI	0.286	0.233
BAI	0.788	0.505

Abbreviations: VFA, visceral fat area; WHR, waist to hip ratio; WHtR, waist to height ratio; WC, waist circumference; BMI, body mass index; BSI, body shape index; BAI, body adiposity index.

Values are the correlation coefficients, and *P* < 0.001 for all analyses.

### Association between hearing thresholds and WHR

Univariate and multivariate linear regression analyses showed that WHR levels were positively associated with four hearing thresholds including PTA and Low-Freq, Mid-Freq, and High-Freq thresholds (Table 5).

**Table 5:** Linear regression analyses of variable hearing thresholds by WHR.

	Univariate		Multivariate	
	Standardized β ± SE	*P*-value*	Standardized β ± SE	*P*-value*
Dep: PTA				
Age	0.569 ± 0.011	<0.001	0.462 ± 0.017	<0.001
DM	0.159 ± 0.488	<0.001	0.015 ± 0.419	0.117
HTN	0.185 ± 0.353	<0.001	0.017 ± 0.309	0.068
WHR	0.492 ± 2.054	<0.001	0.129 ± 3.054	<0.001
Dep: Low-Freq				
Age	0.519 ± 0.010	<0.001	0.413 ± 0.016	<0.001
DM	0.154 ± 0.456	<0.001	0.024 ± 0.407	0.016
HTN	0.209 ± 0.511	<0.001	0.017 ± 0.300	0.078
WHR	0.452 ± 1.964	<0.001	0.124 ± 2.968	<0.001
Dep: Mid-Freq				
Age	0.575 ± 0.012	<0.001	0.474 ± 0.019	<0.001
DM	0.151 ± 0.561	<0.001	0.006 ± 0.480	0.548
HTN	0.185 ± 0.405	<0.001	0.018 ± 0.354	0.061
WHR	0.494 ± 2.358	<0.001	0.123 ± 3.497	<0.001
Dep: High-Freq				
Age	0.662 ± 0.014	<0.001	0.565 ± 0.022	<0.001
DM	0.180 ± 0.707	<0.001	0.014 ± 0.557	0.102
HTN	0.209 ± 0.511	<0.001	0.017 ± 0.411	0.048
WHR	0.557 ± 2.858	<0.001	0.115 ± 4.057	<0.001

*The dependent variable was PTA, Low-Freq, Mid-Freq, or High-Freq, and independent variables were age, DM, HTN, and WHR. Multivariate analysis was performed using age, DM, HTN, and WHR.

Abbreviations: WHR, waist to hip ratio; SE, standard error; Dep, dependent variable; PTA, pure tone average; DM, diabetes mellitus; HTN, hypertension; Low-Freq, low frequency; Mid-Freq, mid-frequency; High-Freq, high-frequency.

**Fig 3:**
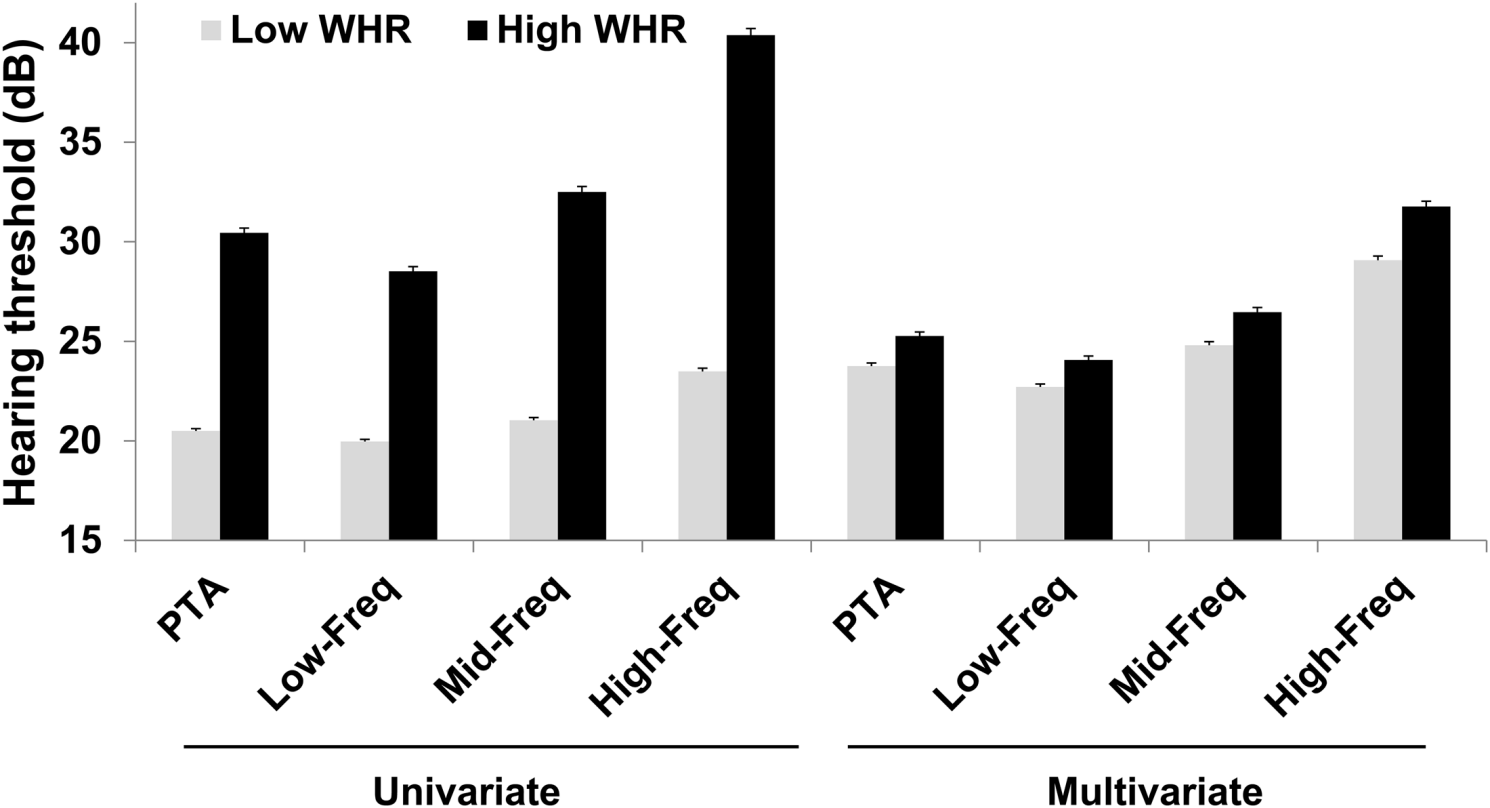
Change in hearing thresholds according to WHR. Multivariate analysis was adjusted for age, diabetes mellitus, hypertension, and WHR (*P* < 0.01 for all univariate and multivariate analyses). Data are expressed as means and standard error. Abbreviations: WHR, waist to hip ratio; PTA, pure tone average; Low-Freq, low frequency; Mid-Freq, mid-frequency; High-Freq, high-frequency.

Differences in hearing thresholds according to the WHR are shown in Fig 3. In univariate analysis, the PTA was 20.5 ± 0.1 dB HL (mean ± standard error) in participants with a WHR lower than or equal to the cutoff value for hearing loss (≤0.9318, low WHR) and 30.4 ± 0.2 dB HL in those with a WHR higher than the cutoff value for hearing loss (>0.9318, high WHR) (*P* < 0.001). The Low-Freq threshold was 20.0 ± 0.1 dB HL in those with a low WHR and 28.5 ± 0.2 dB HL in those with a high WHR (*P* < 0.001). The Mid-Freq threshold was 21.0 ± 0.1 dB HL in those with a low WHR and 32.5 ± 0.3 dB HL in those with a high WHR (*P* < 0.001), and the High-Freq threshold was 23.5 ± 0.2 dB HL in those with a low WHR and 40.4 ± 0.3 dB HL in those with a high WHR (*P* < 0.001). All hearing thresholds in participants with a high WHR were significantly elevated compared to those in participants with a low WHR. In multivariate analysis, the PTA was 23.8 ± 0.16 dB HL (mean ± standard error) in participants with a lower WHR than the cutoff value for hearing loss (≤0.9318, low WHR) and 25.3 ± 0.2 dB HL in those with a higher WHR than the cutoff value for hearing loss (>0.9318, high WHR) (*P* < 0.001). The Low-Freq threshold was 22.7 ± 0.2 dB HL in those with a low WHR and 24.2 ± 0.2 dB HL in those with a high WHR (*P* < 0.001). The Mid-Freq threshold was 24.8 ± 0.2 dB HL in those with a low WHR and 26.5 ± 0.2 dB HL in those with a high WHR (*P* < 0.001). The High-Freq threshold was 29.1 ± 0.2 dB HL in those with a low WHR and 31.8 ± 0.3 dB HL in those with a high WHR (*P* < 0.001). All hearing thresholds in participants with a high WHR were significantly elevated compared to those in participants with a low WHR. In addition, univariate logistic regression analyses showed that the participants with a high WHR had a 5.308–fold increased risk of hearing loss compared with the participants with a low WHR (95% CI, 4.805–5.864; *P* < 0.001). Multivariate analysis revealed that those with a high WHR had a 1.347–fold increased risk of hearing loss compared with the participants with a low WHR (95% CI, 1.178–1.541; *P* < 0.001).

### Comparison between hearing thresholds and the WHR according to age

The numbers of participants in the 40–49, 50–59, 60–69, and ≥70-years-old groups were 2,828, 2,994, 1,534, and 842, respectively, and the WHR in these groups was 0.88 ± 0.03, 0.92 ± 0.04, 0.97 ± 0.04, and 1.00 ± 0.05, respectively. The prevalence of metabolic syndrome in the 40–49, 50–59, 60–69, and ≥70-years-old groups were 5.1%, 14.4%, 27.0%, and 36.0%, respectively (*P* < 0.001), and the prevalence of hearing loss in these groups was 11.0%, 27.9%, 56.3%, and 84.4%, respectively (*P* < 0.001).

All hearing thresholds in participants with a high WHR were significantly elevated compared to those in participants with a low WHR (S1 Table). In addition, univariate logistic regression analyses showed that those with a high WHR in the 40–49, 50–59, 60–69, and ≥70-years-old groups had a 2.042– (95% CI, 1.377–3.028; *P* < 0.001), 1.495– (95% CI, 1.267–1.764; *P* < 0.001), 1.670– (95% CI, 1.289–2.164; *P* < 0.001), and 3.271– (95% CI, 1.688–6.337; *P* < 0.001) fold increased risk of hearing loss compared to participants with a low WHR. Multivariate analyses performed using DM, HTN, and age revealed that those with a high WHR in the 40–49, 50–59, 60–69, and ≥70-years-old groups had a 1.814– (95% CI, 1.205–2.731; *P* = 0.004), 1.231– (95% CI, 1.030–1.470; *P* = 0.022), 1.440– (95% CI, 1.103–1.880; *P* = 0.007), and 3.377– (95% CI, 1.701–6.706; *P* = 0.001) fold increased risk of hearing loss compared to participants with a low WHR.

## Discussion

In this study, we investigated the predictive effect of WHR compared with WHtR, WC, BMI, BSI, and BAI with respect to metabolic syndrome or hearing loss. We found that WHR had the highest AUC for prediction of metabolic syndrome or hearing loss and was highly correlated with VFA measured by bioimpedance analysis and numbers of metabolic syndrome components. A High WHR was associated with elevated hearing thresholds including the PTA and Low-Freq, Mid-Freq, and High-Freq thresholds. This result suggests that WHR in Asian women is associated with the prevalence of hearing impairment results from the metabolic syndrome.

First, we established the best predictive index for predicting metabolic syndrome or hearing loss among WHR, WHtR, WC, BMI, BSI, and BAI. Analyses using AUROC showed the AUCs for predicting metabolic syndrome or hearing loss among these indices, and we identified cutoff values (0.939 for metabolic syndrome and 0.932 for hearing loss. WHR had the highest correlation coefficient for numbers of metabolic syndrome components and high AUC values for each metabolic syndrome component. In addition, we investigated the association between VFA and WHR. VFA as an important metabolic parameter was measured using bioimpedance analysis. WHR was highly correlated with VFA (*r* = 0.901). These results suggest that WHR is a good indicator for predicting metabolic syndrome or hearing loss.

Second, we performed additional analyses regarding the association between hearing impairment and WHR. Univariate and multivariate linear regression analyses showed that PTA and Low-Freq, Mid-Freq, and High-Freq thresholds were positively associated with WHR as a continuous variable. When participants were divided into two categorical groups according to the cutoff value for hearing loss, participants with a high WHR had significantly elevated hearing thresholds compared to those with a low WHR. Logistic regression analysis also showed that a high WHR was associated with an increased risk of hearing loss. These results suggest that WHR may be associated with the prevalence of hearing loss through prediction of metabolic disturbances.

The best predictor for metabolic syndrome among the anthropometric indices still remains unsolved. BMI is a classical indicator for obesity. However, BMI does not differentiate between fat mass and fat-free mass and cannot evaluate the distribution of fat. Visceral adipose tissue is more cellular, vascular, and innervated and contains a larger number of inflammatory and immune cells than subcutaneous adipose tissue [19]. WC was introduced as an alternative to BMI, but that is sensitive to body size as well as fat distribution [14]. Therefore, surrogates such as WHR, WHtR, BSI, or BAI were introduced. Some studies revealed that these indicators adjusted for body size or fat distribution are better discriminators of metabolic problems compared with BMI or WC as classical indicators [13,14,20,21]. However, there were conflicting results regarding which indicators were better according to gender and ethnicity. Ko et al. investigated the optimal obesity index in a Korean population and demonstrated that WHR is better discriminator than BMI, WHtR, and WC [22]. Similarly, the present study shows that WHR is the best predictor for metabolic syndrome.

Except for BMI and WC, data for the relationships between anthropometric indices and hearing loss are scant. Although many studies have investigated the association between BMI and hearing, results were conflicting [4,5,7,23,24]. Some cross-sectional studies showed an association between hearing and WC or WHR [5–8]. Some authors showed a positive association between WC and hearing loss, but surrogates adjusted for body size may have more accuracy than WC [5,7]. Two studies showed an association between WHR and hearing [6,8]. However, Marron et al. [8] enrolled only a Swedish population and El-Mansoury et al. [6] enrolled only participants with Turner syndrome. In addition, there are few comparative studies using these indices. The present study enrolled Asian women and compared variable indices for hearing loss. Our results suggest that WHR was highly associated with hearing loss or hearing thresholds, and these associations may be due to correlation between WHR and metabolic disturbances.

We have focused on age-related sensorineural hearing loss. Many studies have evaluated the risk factors for, or the prevalence of age-related hearing loss, although no definite age criteria have been established. Therefore, since definite criteria for age-related hearing loss would not be case-sensitive, we set the age criteria for women as ≥40 years old in this study. Some studies included participants ≥20 years old, whilst others only included participants who were ≥50 years old. Age-related sensorineural hearing loss starts slowly around the fifth decade, and if mainly young participants are included, the prevalence of hearing loss could be underestimated and the study findings could be influenced by the effect of sex hormones [25]. If mainly older participants are included, smaller study cohorts and the presence of comorbidities are likely to be study limitations. In addition, the primary objective of the present study was to evaluate the relationship between WHR and metabolic syndrome or hearing impairment. Non-elderly participants with metabolic disorders may develop hearing loss, and thus a non-elderly population was included in the present study, and a multivariate analysis adjusted for age was used to exclude age-related factors.

In both humans and other mammals, DM is associated with thickening of the basement membrane or atrophy of the capillaries in the stria vascularis of the cochlea, which is similar to DM induced microangiopathy, and thus individuals with DM may be at greater risk of hearing loss [26,27]. However, there have been conflicting results regarding the association between hearing loss and DM. A number of studies found a positive association between the two variables [28,29]. However, Dalton et al. demonstrated a positive, but weak association between DM and hearing loss [30]. Mitchell et al. showed a negative association with prevalent hearing loss of DM, but a positive association with incident hearing loss of DM [31]. Other studies did not show an association between DM and hearing loss [32,33]. A recent meta-analysis concluded that mild hearing loss is more prevalent in participants with DM, which has greater clinical relevance in individuals who are older or who have had DM over a longer duration [34].

The relationship between hearing loss and HTN is another important issue. Although the mechanisms that underlie this relationship are poorly understood, a number of studies have indicated that abnormalities of the stria vascularis may play a key role in HTN-related hearing loss [35,36]. Previous studies have demonstrated a positive association between HTN and hearing loss, although Rosenhall et al. found that there was an inconsistent association between these variables [37–40]. HTN often coexists with other cardiovascular risk factors such as dyslipidemia or DM, making it more difficult to determine whether hearing loss is definitely cause by HTN, and to evaluate the associations between these other variables. Most previous studies found that HTN was associated with high-frequency hearing impairment, and likewise, in this study, we found a positive association between these variables.

The incidence of both metabolic syndrome and hearing loss increases considerably with age, making it an important confounding factor [41]. We also found that there was a higher prevalence of metabolic syndrome, hearing loss, and lower WHR with increasing age. Although hearing loss is not actually associated with WHR, there may appear to be a positive association as both are related to age. Therefore, we performed multivariate analyses that included age as a covariate, as well as age stratification analyses. These multivariate and subgroup analyses can reduce the confounding effect of age for hearing loss, and the present study revealed a positive association between WHR and hearing thresholds or hearing loss.

There are differences between the sexes in the predictive value of central obesity for metabolic disease or hearing loss. Some authors suggest that sex difference may be due to plasma adiponectin and estrogen levels [16,42,43]. Indices related to central obesity may be more predictive in women than in men and the present study enrolled only women.

The present study has a number of limitations. First, it is retrospective in nature and cannot confirm the causality between WHR and metabolic syndrome or hearing impairment. Second, habitual parameters such as alcohol consumption and smoking, and history of exposure to noise such as occupational or explosive noise were not evaluated in the present study. Third, the participants volunteered for screening. All of the enrolled patients were analyzed and most lived in an urban area. In addition, we did not use power calculation to determine the sample size because this was not a prospective study. Therefore, the participants may not be representative of the general Korean population. However, the impact of these limitations would be reduced by the large sample size in this study. Further prospective analysis, including follow-up data, is needed to confirm the possible strong correlations between variables.

In summary, our results demonstrated that WHR may be a surrogate marker for predicting the risk of hearing loss resulting from metabolic syndrome.

## Supporting Information

S1 Fig Histogram showing the age distribution of study participants. (TIF)

S2 Fig The numbers of participants according to referral area in the present study. There were 5,324 participants in Daegu Metropotian City, 2,243 in Gyeongsangbuk Province, 451 in Gyeongsangnam Province, 24 in Seoul Metropolitan City, 22 in Gyeonggi Province, 18 in Ulsan Metropolitan City, 11 in Pusan Metropolitan City, 11 in Kangwon Province, 8 in Chungcheongnam Province, 6 in Chungcheongbuk Province, 5 in Daejon Metropolitan City, 2 in Jeollanam Province, 2 in Jeollabuk Province, 2 in Jeju Province, 1 in Inchon Metropolitan City, 1 in Kwangju Metropolitan City, and 13 in other countries. No data was available regarding the place of residence for 54 participants.
(TIF)

S1 Table Comparison of hearing thresholds according to waist to hip ratio in different age groups. (DOC)

S1 Text List of abbreviations. (DOC)
