# Supplementary figures and images for: Comparison of Various Anthropometric Indices as Risk Factors for Hearing Impairment in Asian Women
Source: PLoS One. 2015 Nov 17;10(11):e0143119. doi: 10.1371/journal.pone.0143119 (PMC4648514; doi:10.1371/journal.pone.0143119)

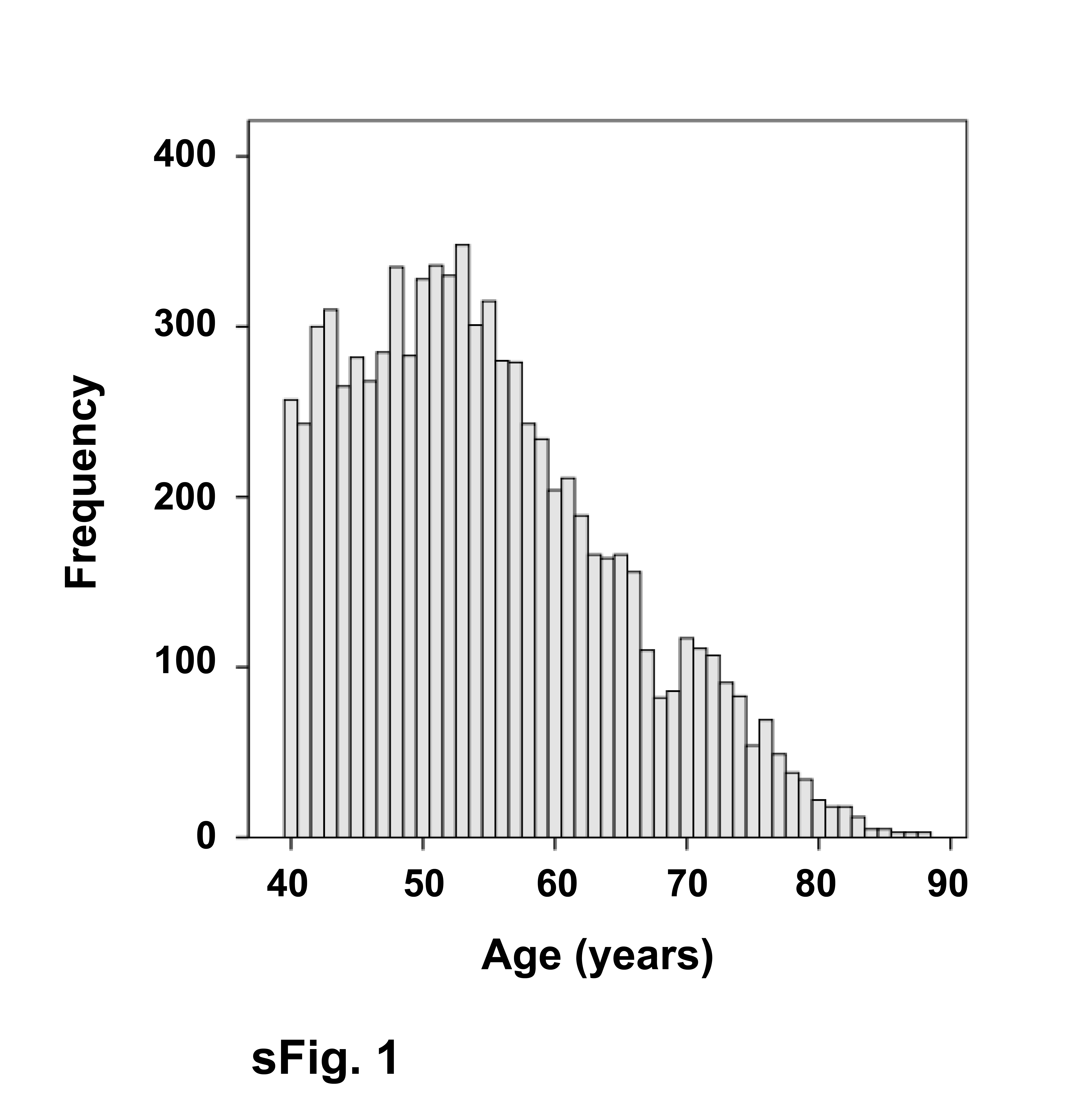

Supplement: S1 Fig — (TIF) [file pone.0143119.s001.tif]

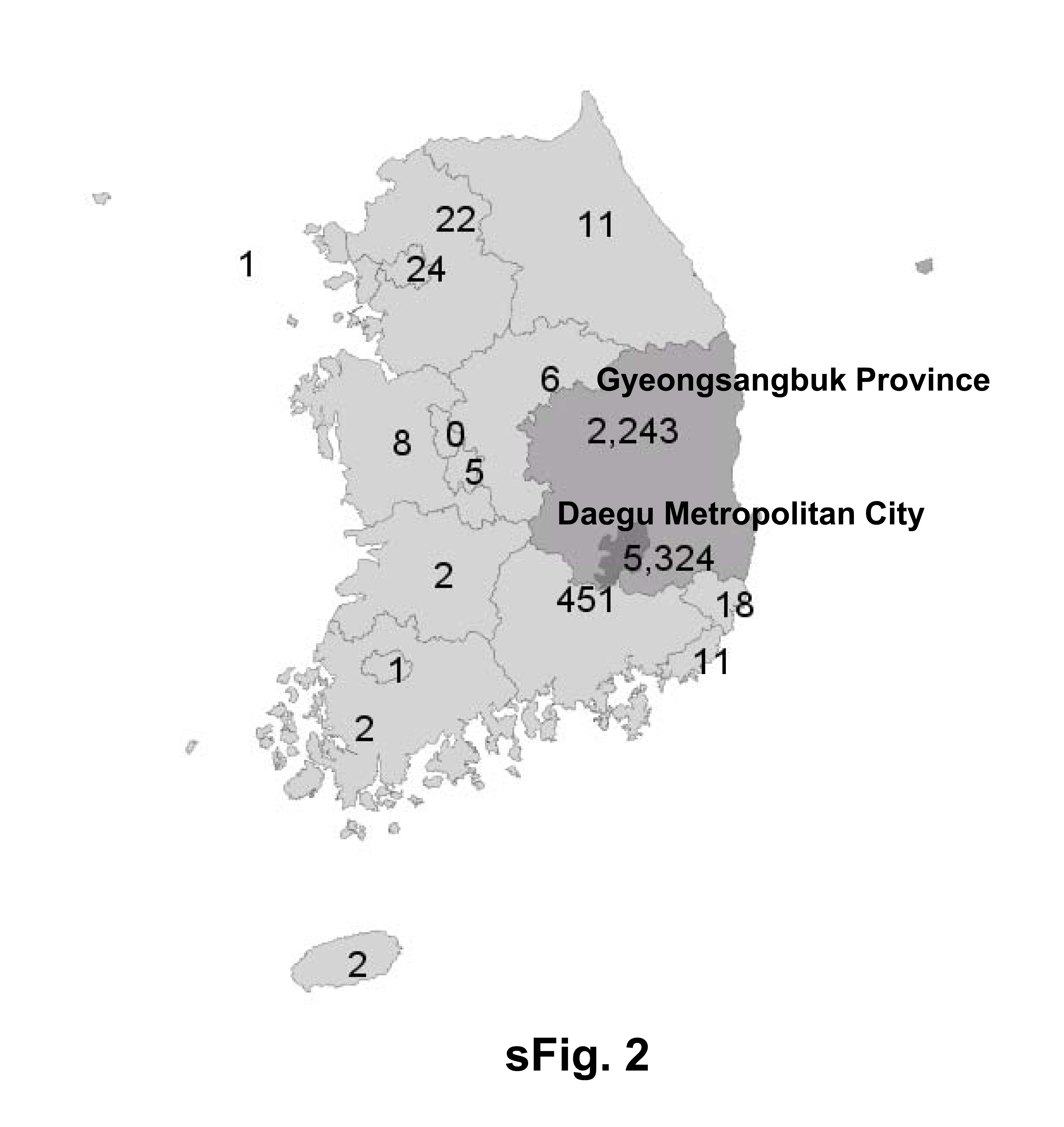

Supplement: S2 Fig — There were 5,324 participants in Daegu Metropotian City, 2,243 in Gyeongsangbuk Province, 451 in Gyeongsangnam Province, 24 in Seoul Metropolitan City, 22 in Gyeonggi Province, 18 in Ulsan Metropolitan City, 11 in Pusan Metropolitan City, 11 in Kangwon Province, 8 in Chungcheongnam Province, 6 in Chungcheongbuk Province, 5 in Daejon Metropolitan City, 2 in Jeollanam Province, 2 in Jeollabuk Province, 2 in Jeju Province, 1 in Inchon Metropolitan City, 1 in Kwangju Metropolitan City, and 13 in other countries. No data was available regarding the place of residence for 54 participants. (TIF) [file pone.0143119.s002.tif]
