# Supplementary material for: Comparison of Various Anthropometric Indices as Risk Factors for Hearing Impairment in Asian Women
Source: PLoS One. 2015 Nov 17;10(11):e0143119. doi: 10.1371/journal.pone.0143119 (PMC4648514; doi:10.1371/journal.pone.0143119)
Supplement: S1 Table — (DOC) [file pone.0143119.s003.doc]

| **Univariate** | **Low-Freq** | |  | **Mid-Freq** | |  | **High-Freq** | |  | **PTA** | |
| --- | --- | --- | --- | --- | --- | --- | --- | --- | --- | --- | --- |
|  | Mean ± SE (dB HL) | *P* |  | Mean ± SE (dB HL) | *P* |  | Mean ± SE (dB HL) | *P* |  | Mean ± SE (dB HL) | *P* |
| 40**–**49 years |  | <0.001 |  |  | <0.001 |  |  | <0.001 |  |  | <0.001 |
| Low WHR | 18.3 ± 0.1 |  |  | 18.6 ± 0.2 |  |  | 19.7 ± 0.2 |  |  | 18.4 ± 0.1 |  |
| High WHR | 20.3 ± 0.6 |  |  | 20.9 ± 0.6 |  |  | 23.7 ± 0.8 |  |  | 20.6 ± 0.6 |  |
| 50**–**59 years |  | <0.001 |  |  | <0.001 |  |  | <0.001 |  |  | <0.001 |
| Low WHR | 21.3 ± 0.2 |  |  | 23.0 ± 0.2 |  |  | 26.4 ± 0.3 |  |  | 22.2 ± 0.2 |  |
| High WHR | 23.2 ± 0.3 |  |  | 25.5 ± 0.3 |  |  | 30.1 ± 0.4 |  |  | 24.3 ± 0.3 |  |
| 60**–**69 years |  | <0.001 |  |  | <0.001 |  |  | <0.001 |  |  | <0.001 |
| Low WHR | 24.8 ± 0.6 |  |  | 28.2 ± 0.7 |  |  | 34.6 ± 0.9 |  |  | 26.5 ± 0.6 |  |
| High WHR | 28.6 ± 0.4 |  |  | 32.5 ± 0.4 |  |  | 41.3 ± 0.5 |  |  | 30.5 ± 0.4 |  |
| ≥70 years |  | 0.002 |  |  | 0.005 |  |  | 0.009 |  |  | 0.002 |
| Low WHR | 29.8 ± 1.9 |  |  | 36.5 ± 2.2 |  |  | 48.9 ± 2.5 |  |  | 33.2 ± 2.0 |  |
| High WHR | 37.1 ± 0.5 |  |  | 44.1 ± 0.6 |  |  | 56.0 ± 0.6 |  |  | 40.5 ± 0.5 |  |
| **Multivariate** |  |  |  |  |  |  |  |  |  |  |  |
| 40**–**49 years |  | 0.006 |  |  | 0.007 |  |  | 0.000 |  |  | 0.004 |
| Low WHR | 18.3 ± 0.1 |  |  | 18.6 ± 0.1 |  |  | 19.8 ± 0.2 |  |  | 18.4 ± 0.1 |  |
| High WHR | 19.7 ± 0.5 |  |  | 20.2 ± 0.6 |  |  | 22.6 ± 0.7 |  |  | 20.0 ± 0.5 |  |
| 50**–**59 years |  | 0.001 |  |  | 0.002 |  |  | <0.001 |  |  | 0.001 |
| Low WHR | 21.6 ± 0.2 |  |  | 23.4 ± 0.2 |  |  | 27.0 ± 0.3 |  |  | 22.5 ± 0.2 |  |
| High WHR | 22.7 ± 0.3 |  |  | 24.7 ± 0.3 |  |  | 28.9 ± 0.4 |  |  | 23.7 ± 0.3 |  |
| 60**–**69 years |  | <0.001 |  |  | <0.001 |  |  | <0.001 |  |  | <0.001 |
| Low WHR | 25.2 ± 0.7 |  |  | 28.8 ± 0.8 |  |  | 36.2 ± 1.0 |  |  | 27.0 ± 0.7 |  |
| High WHR | 28.2 ± 0.3 |  |  | 32.2 ± 0.4 |  |  | 41.0 ± 0.5 |  |  | 30.2 ± 0.3 |  |
| ≥70 years |  | 0.001 |  |  | 0.004 |  |  | 0.008 |  |  | 0.002 |
| Low WHR | 29.6 ± 2.2 |  |  | 36.4 ± 2.5 |  |  | 49.0 ± 2.5 |  |  | 33.0 ± 2.3 |  |
| High WHR | 36.9 ± 0.5 |  |  | 43.8 ± 0.6 |  |  | 56.0 ± 0.6 |  |  | 40.3 ± 0.5 |  |

*The dependent variable was PTA, Low-Freq, Mid-Freq, or High-Freq, and multivariate analysis was performed using age, DM, HTN, and WHR.

Abbreviations: WHR, waist to hip ratio; SE, standard error; Low-Freq, low frequency; Mid-Freq, mid-frequency; High-Freq, high-frequency; PTA, pure tone average; DM, diabetes mellitus; HTN, hypertension.
