# Supplementary material for: Comparison of Various Anthropometric Indices as Risk Factors for Hearing Impairment in Asian Women
Source: PLoS One. 2015 Nov 17;10(11):e0143119. doi: 10.1371/journal.pone.0143119 (PMC4648514; doi:10.1371/journal.pone.0143119)
Supplement: S1 Text — (DOC) [file pone.0143119.s004.doc]

**Supporting Information: List of abbreviations**

WHR, waist to hip ratio

HTN, hypertension

WC, waist circumference

HDL, high-density lipoprotein

BMI, body mass index

WHtR, waist to height ratio

DM, diabetes mellitus

BSI, body shape index

BAI, body adiposity index

VFA, visceral fat area

PTA, pure tone average

AUROC, area under the receiver operating characteristic curve

AUC, area under the curve

FG, fasting glucose

BP, blood pressure

TG, triglyceride

HDL-C, high-density lipoprotein cholesterol

CI, Confidence interval
